# Integrating the Water Planetary Boundary With Water Management From Local to Global Scales

**DOI:** 10.1029/2019EF001377

**Published:** 2020-02-13

**Authors:** Samuel C. Zipper, Fernando Jaramillo, Lan Wang‐Erlandsson, Sarah E. Cornell, Tom Gleeson, Miina Porkka, Tiina Häyhä, Anne‐Sophie Crépin, Ingo Fetzer, Dieter Gerten, Holger Hoff, Nathanial Matthews, Constanza Ricaurte‐Villota, Matti Kummu, Yoshihide Wada, Line Gordon

**Affiliations:** ^1^ Kansas Geological Survey University of Kansas Lawrence KS USA; ^2^ Department of Civil Engineering University of Victoria Victoria British Columbia Canada; ^3^ Department of Physical Geography Stockholm University Stockholm Sweden; ^4^ Baltic Sea Centre Stockholm University Stockholm Sweden; ^5^ Stockholm Resilience Centre Stockholm University Stockholm Sweden; ^6^ Bolin Centre for Climate Research Stockholm University Stockholm Sweden; ^7^ International Institute for Applied Systems Analysis Laxenburg Austria; ^8^ Beijer Institute of Ecological Economics Royal Swedish Academy of Sciences Stockholm Sweden; ^9^ Potsdam Institute for Climate Impact Research, Member of the Leibniz Association Potsdam Germany; ^10^ Department of Geography Humboldt‐Universität zu Berlin Berlin Germany; ^11^ Stockholm Environment Institute Stockholm Sweden; ^12^ Global Resilience Partnership Stockholm Sweden; ^13^ Instituto de Investigaciones Marinas y Costeras “José Benito Vives de Andreis” Santa Marta Colombia; ^14^ Water and Development Research Group Aalto University Espoo Finland

**Keywords:** water management, Earth Systems, cross‐scale, water cycle, Anthropocene, planetary boundaries

## Abstract

The planetary boundaries framework defines the “safe operating space for humanity” represented by nine global processes that can destabilize the Earth System if perturbed. The water planetary boundary attempts to provide a global limit to anthropogenic water cycle modifications, but it has been challenging to translate and apply it to the regional and local scales at which water problems and management typically occur. We develop a cross‐scale approach by which the water planetary boundary could guide sustainable water management and governance at subglobal contexts defined by physical features (e.g., watershed or aquifer), political borders (e.g., city, nation, or group of nations), or commercial entities (e.g., corporation, trade group, or financial institution). The application of the water planetary boundary at these subglobal contexts occurs via two approaches: (i) calculating *fair shares*, in which local water cycle modifications are compared to that context's allocation of the global safe operating space, taking into account biophysical, socioeconomic, and ethical considerations; and (ii) defining a *local safe operating space,* in which interactions between water stores and Earth System components are used to define local boundaries required for sustaining the local water system in stable conditions, which we demonstrate with a case study of the Cienaga Grande de Santa Marta wetlands in Colombia. By harmonizing these two approaches, the water planetary boundary can ensure that water cycle modifications remain within both local and global boundaries and complement existing water management and governance approaches.

## Key Points

- Local and regional water management responds to and is influenced by global water cycle processes
- The planetary boundaries framework is useful for reconciling local and global water sustainability goals
- The framework can be applied at scales including nations, watersheds, aquifers, or commercial entities

## 1 Local Water Resources and Earth System Stability

Water is fundamental to Earth System functioning and human society. Due to the central role of water for maintaining global biosphere integrity, regulating climate, and mediating carbon and nutrient cycling, changes to the water cycle can propagate through the Earth System and disrupt processes interacting across numerous scales. For example, land use change in one setting can alter evapotranspiration and lead to precipitation change downwind (Wang‐Erlandsson et al., 2018). Self‐amplifying land‐water interactions mean that deforestation may lead to regional forest dieback in areas such as the Amazon (Zemp et al., 2017) with potential cascading impacts on Earth System stability as a whole (Steffen et al., 2018). In addition to physical processes, socioeconomic factors external to a watershed can impact local hydrological conditions: Agriculture, by far the largest user of freshwater, is driven by global socioeconomic decisions as crops are shipped all over the world (Hoekstra & Mekonnen, 2012; Jaramillo & Destouni, 2015). Anthropogenic climate change, a global challenge, also has diverse impacts on local water systems (Cook et al., 2018; Pfahl et al., 2017). In other words, the local water cycle is shaped by global processes and local hydrological changes can have global consequences.

This emerging understanding of interconnections between local and global water systems requires integrated management and governance strategies across scales (Biermann et al., 2012; Sivapalan et al., 2014). However, developing generalizable understanding of the spatiotemporal scales spanned by the water cycle has been a longstanding challenge in hydrology, water management, and at their intersection (Blöschl et al., 2019; Blöschl & Sivapalan, 1995; Daniell & Barreteau, 2014; Klemeš, 1983). In particular, recent work has identified translating understanding of coupled human and natural systems across scales as a key future research priority to provide management‐relevant science (Konar et al., 2016; Kramer et al., 2017). While sociohydrology has been suggested as a potential tool to bridge the gaps between watershed‐scale and global‐scale water management (Di Baldassarre et al., 2019), specific approaches for integrating global water sustainability targets with local water management remain lacking.

**Figure 1:**
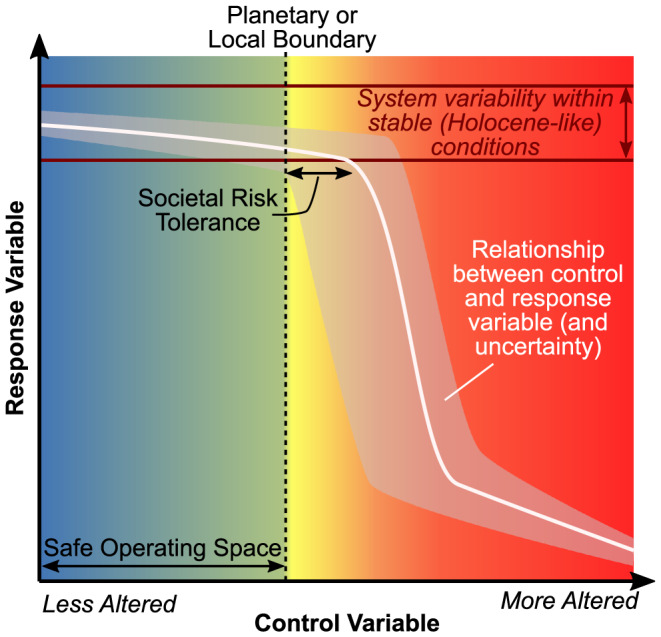
A planetary or local boundary (dashed line) is set where the system shifts from stable to possibly destabilized conditions in response to change in the control variable. A precautionary approach takes into account the system variability of the response variable (dark red horizontal lines), scientific and systemic uncertainty about the relationship between the control and response variables (zoning around the white curve), and the societal tolerance of risk (setback of boundary from threshold). The relationship between the control and response variables shown here is just one possible relationship, and these relationships are not necessarily threshold type or even monotonic.

The planetary boundaries framework, introduced by Rockström et al. (2009) and further elaborated by Steffen et al. (2015), offers one approach to bring a global perspective to local water management (Konar et al., 2016). The planetary boundaries framework identifies nine boundaries representing critical Earth System processes. Transgressing these boundaries substantially increases the risks of irreversibly destabilizing these processes. For most of these processes, a quantifiable *control variable* has been suggested, which may cause some *response variable* to destabilize, either alone or through interactions with other Earth System processes (Figure 1; Rockström et al., 2009; Steffen et al., 2015). For effective boundary setting, the control variable should be quantifiable and subject to influence by human actions, while the response variable should describe Earth's stable conditions and be influenced by the control variable (Gleeson et al., 2020). The boundary value of each control variable is set some distance upstream from departure of the response variable from stable conditions, typically at the lower end of uncertainty due to systemic and/or scientific factors (Figure 1). A given control‐response variable relationship and corresponding boundary value may not be static through time but subject to change due to interactions with other planetary boundaries as well as process lags and hysteretic effects. The safe operating space bounded by the nine planetary boundaries describes the Holocene‐like Earth System conditions, which so far are the only ones in which human civilization has thrived.

Regarding water, which is our focus here, the planetary boundary for freshwater use was originally based on the amount of freshwater that could be withdrawn while maintaining rivers' environmental flow requirements globally (Gerten et al., 2013; Steffen et al., 2015). Recently, Gleeson et al. (2019, 2020) proposed to distinguish six water sub‐boundaries relating to the major stores of freshwater to more holistically represent the various functions of water in maintaining Earth System stability (Table 1). Environmental flow requirements are retained in this approach as the surface water sub‐boundary, together with new water planetary sub‐boundaries for frozen water, groundwater, soil moisture, and two sub‐boundaries for different aspects of atmospheric water. Gleeson et al. (2019, 2020) suggested potential control and response variables for these new sub‐boundaries (Table 1), but significant work remains to select and evaluate appropriate variables and boundary values. To provide a sound societal relevance for these efforts, it is necessary to first determine whether the water planetary boundary can be meaningfully integrated with existing water management approaches.

**Table 1:** Proposed Water Planetary Sub‐boundaries From Gleeson et al. (2019, 2020) That Correspond to Water Stores and Functions

Water store	Core function of this water store in the Earth System	Possible response variable(s)	Possible control variable(s)	Cross‐scale interaction not considered in traditional water management approaches
Atmospheric water	Hydroclimatic regulation	Climate pattern stability or land‐atmosphere coupling	Land area with evaporation change	Changes in precipitation due to upwind changes in land use or irrigation (Keys et al., 2017, 2018)
	Hydroecological regulation	Terrestrial biosphere integrity	Land area with precipitation change	
Soil moisture	Hydroclimatic regulation	Carbon uptake or net primary productivity	Global root zone storage capacity	Trade‐offs between global CO_2_ budget and local water availability (Heck, Gerten, et al., 2018)
Surface water	Hydroecological regulation	Aquatic biosphere integrity	Watersheds or total river length within environmental flow limits	Importance of local aquatic systems to global biodiversity pool (Mace et al., 2014)
Groundwater	Storage	Terrestrial or aquatic biosphere integrity	Watersheds with sufficient low flows	Groundwater coupling with global climate system (Cuthbert et al., 2019)
Frozen water	Storage	Sea level rise	Volume of ice melt	Local responsibility for global sea level rise (Hardy & Nuse, 2016) and knock‐on impacts on other planetary boundaries (Crépin et al., 2017)

*Note*. Control variables are not defined.

Since there are no planetary‐scale water management and governance institutions (Biermann et al., 2012), the water planetary boundary needs to be translated to the local and regional scales where water management and governance operate (Cambridge Institute for Sustainability Leadership [CISL], 2019; Konar et al., 2016). In this study, we address three questions necessary to integrate local water management and governance with global water sustainability:

1. How can global‐scale values be meaningfully disaggregated to the diverse spatial scales at which water management and governance occurs such as watersheds, nations, and commercial entities?
2. How does the planetary boundaries framework complement existing water management approaches at each of these spatial scales?
3. What scientific questions need to be addressed to move the planetary boundaries framework forward as a potential water management approach?

**Figure 2:**
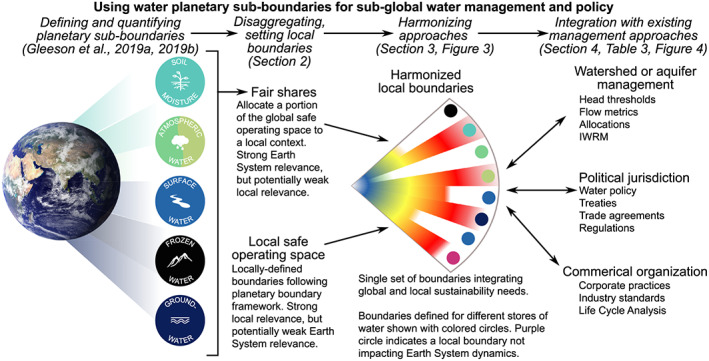
Steps to translate water's planetary role in Earth System dynamics to local management and governance. In the harmonized approach, colored circles indicate a water sub‐boundary corresponding to a specific store of water and the red‐yellow‐blue color gradient corresponds with the current position of the control variable with respect to the boundary, as in Figure 1. The fair shares approach will subdivide each water planetary sub‐boundary into the local context. The local safe operating space approach may have local boundaries corresponding to all or some of the sub‐boundaries, as well as additional locally relevant boundaries that may not have an impact on Earth System function. Further details about each step are provided in the text sections and figures referenced at the top of the figure.

We develop a flexible approach to applying the water planetary boundary across different scales and jurisdictions, in order to complement existing management and governance approaches by accounting for interactions across traditional water system borders and scales and for relationships among different components of the Earth System (Figure 2). In section 2, we synthesize previous literature on subglobal use of the planetary boundaries framework to classify and explore two approaches, *fair shares* and *local safe operating space*, in order to identify strengths, weaknesses, and principles for effective implementation of each approach. In section 3, we present a methodology to integrate these two approaches to calculate harmonized boundaries with strong connections to both global and local water sustainability. We conclude that the water planetary boundary can be used in subglobal domains defined using physical features (e.g., watershed or aquifer management), political borders (e.g., a city, nation, or group of nations), or commercial entities (e.g., companies, industries, or trade groups operating within or across national borders). For brevity, we will use the term *local contexts* to refer interchangeably to any of these subglobal applications. As one example, we demonstrate how the water planetary boundary can be used in the context of a degraded hydrological system, the Cienaga Grande de Santa Marta wetland complex in Colombia (Box 1). Furthermore, because the water sub‐boundaries highlight key interactions between water cycle change, climate change, and land system change, we find that the term *water management* needs to be broadened to refer to any type of management of Earth System processes that have significant interactions with the water cycle, not only management of liquid water in surface water and aquifers.

## 2 Principles for Using the Planetary Boundary in Subglobal Water Management

### 2.1 Fair Shares Approach

The *fair shares* approach is a top‐down approach that treats the planetary boundary value of the control variable as a global safe operating space “budget” and then allocates a portion of that global safe operating space to a given local context. The fair shares approach has been used in diverse local contexts including nations, cities, companies, and industries (Table S1 in the supporting information). This approach has strong Earth System relevance because it is directly connected to water's functions that define the planetary boundaries. This also means it may have limited local relevance because the globally defined control variable may not be the most effective descriptor or authoritative guidance for modifications of the water system in some local contexts. If global fair shares exceed socially and ecologically important thresholds defining the local safe operating space (discussed in section 2.2), local decision makers would intervene in the water system well before the global boundary is reached, and therefore, the fair shares boundaries would not be a relevant local consideration.

The fair shares approach requires three steps: (1) setting the planetary boundary value(s); (2) allocating a fraction of the global safe operating space to a local context; and (3) comparing current performance to the allocation (i.e., to the local fair share) for each control variable.

#### 2.1.1 Setting the Planetary Boundary

The first step in operationalizing the fair shares approach is defining the safe operating space by determining values for each of the water planetary sub‐boundaries. While recent work has proposed new planetary sub‐boundaries for different stores of water in the Earth System (Gleeson et al., 2019, 2020), previous efforts to operationalize the planetary boundaries framework using the fair shares approach have all used the control variable and estimated boundary values from Rockström et al. (2009) and Steffen et al. (2015) (Table S1). In the case of water, this means that the planetary boundary is typically taken as 4,000 km^3^ year^−1^ of consumptive blue water use. To better account for the ecological impacts of human water use, Gerten et al. (2013) suggested a spatially explicit quantification of environmental flow requirements to focus on the impacts of freshwater use for local biosphere integrity. Using this approach, they calculated monthly environmental flow requirements for all surface water globally, which resulted in a boundary between 1,100 and 4,500 km^3^ year^−1^. Reframing the water planetary boundary for different stores of water as proposed by Gleeson et al. (2019, 2020) improves the ability of the fair shares approach to accurately represent water's role in Earth System dynamics, and therefore, we suggest that future work on the fair shares approach should shift to these new control and response variables (Table 1).

#### 2.1.2 Allocating the Global Safe Operating Space to Local Contexts

The fair shares approach requires allocating the global safe operating space to subglobal scales and entities. For commercial organizations, economic indicators such as a corporation's global market share are typically used (Ryberg et al., 2018; Sandin et al., 2015). For political contexts (e.g., nations), allocation is most often implemented using a per capita approach in which the global value of the control variable of the planetary boundary is apportioned based on the number of people living in that nation (Dao et al., 2015; Hoff et al., 2014; Nykvist et al., 2013; O'Neill et al., 2018). The major flaw of per capita allocation is that it allocates a larger portion of the global safe operating space to more populous nations, without taking into account local hydrological factors or people's capacity to respond to environmental challenges (Häyhä et al., 2018). Alternative allocation principles, for example, based on equality, rights, socioeconomic capacity, and the responsibility of different groups of people, could be used instead (see Häyhä et al., 2016; Lucas & Wilting, 2018; or literature on allocating greenhouse gas emissions reviewed in Zhou & Wang, 2016).

Choosing among allocation approaches requires ethical and political decisions that account for local differences in the contribution to global environmental challenges, the ability to respond to them, and differing definitions of “fair” among stakeholders (Biermann, 2012; Häyhä et al., 2016). For instance, the suggested frozen water sub‐boundary quantifies global ice melt (Table 1), which is strongly driven by anthropogenic climate change; however, the countries that contribute most to climate change are not the same countries that feel the strongest impacts, posing an ethical challenge (Althor et al., 2016; Biemans et al., 2019). Addressing ethical issues is particularly challenging because different definitions of equity and resulting allocation approaches can lead to substantial differences in what is estimated as a fair share of the global safe operating space (Häyhä et al., 2016; Ryberg et al., 2018). Regardless of the allocation approach used, any fair shares approach will also be sensitive to the estimated planetary boundary value, which may include substantial uncertainty that must be accounted for and communicated as part of the fair shares allocation.

#### 2.1.3 Comparing Current Performance to the Allocation for Each Control Variable

Previous planetary boundaries applications have used both production‐based and consumption‐based approaches to calculate a local context's performance relative to the allocation of a local fair share (Table S1). A production‐based approach considers impacts of the production of goods and services on the water cycle only within the local context, such as within a nation. However, many environmental impacts are partially externalized via trade (Dalin et al., 2017; Marston et al., 2015; Wiedmann & Lenzen, 2018) and/or felt in locations distant from where the water use occurs (e.g., transboundary effects; Munia et al., 2016; Veldkamp et al., 2017). A consumption‐based approach therefore considers the global impacts on the water cycle associated with the water used to supply all the goods and services consumed within the local context. This is known as embedded or virtual water, and there is growing consensus that it should be accounted for in sustainable resource‐use decision making (D'Odorico et al., 2019). Geographically explicit approaches such as water footprints can be used for consumption‐based quantification (Mekonnen & Hoekstra, 2011; Vanham et al., 2019). Companies and industries, which operate across borders, frequently use consumption‐based life cycle analyses, which account for the various materials and impacts of processes required in complex global supply chains, and have begun to integrate the planetary boundaries framework as a lens to interpret the results of life cycle analyses (Brejnrod et al., 2017; Wolff et al., 2017). Life cycle analyses can also use the water footprint approach to quantify the performance of a product, company, or supply chain for water impacts all over the world (Chapagain et al., 2005; Kounina et al., 2013; Pfister et al., 2009). The water footprint can be adjusted based on water scarcity at the location of production, that is, where the consumptive use takes place, to more directly reflect environmental impacts (Ridoutt & Pfister, 2010).

The new control variables of Gleeson et al. (2019, 2020) will require the development of novel approaches for consumption‐based quantification. As an example, they suggest that the control variable for atmospheric water's role in hydroclimatic regulation may be the degree of human‐caused change in evapotranspiration, for instance, due to land use change and water use. The water footprint concept described above offers a way to link different changes in evapotranspiration to specific local actions such as land use change or irrigation (Schyns et al., 2019). Land use footprints and indirect land use change metrics (Searchinger et al., 2008; Weinzettel et al., 2013) can likely be adapted to meet the needs of the fair shares approach for assessing subglobal responsibility. However, these sorts of attribution studies are disputed due to the difficulties involved in tracing how national policies propagate through the global economic system to influence land use (Mathews & Tan, 2009; Zilberman, 2017).

### 2.2 Local Safe Operating Space Approach

The *local safe operating space* approach is a bottom‐up approach that uses the principles of the planetary boundaries framework to generate locally meaningful control variables, response variables, and boundary values defining the local stable conditions of the water system (Figure 1). Local variables may or may not be the same as the planetary boundary control and response variables, because the drivers of hydrological stability in local water systems can differ from drivers of stability at the Earth System scale. This approach allows stakeholders and water managers working on a specific region to define safe operating spaces that have a strong relevance to the local socioenvironmental system and can inform efficient water management interventions. However, local safe operating spaces have potentially weak relevance at the Earth System scale due to their local focus.

The local safe operating space approach also typically contains three steps: (1) defining locally meaningful control and response variables, which may differ from the variables used for the planetary boundaries; (2) setting boundary values that define the local safe operating space; and (3) quantifying the current state of each control variable.

#### 2.2.1 Defining the Control and Response Variables

The local safe operating space approach focuses on the local water system and does not have an explicit relationship to Earth System stability (though local effects may scale up to affect Earth System stability). The definition of locally relevant control and response variables should be based on the biophysical and socioeconomic limits of the local water system, which may already be identified in thresholds or allocations from existing water management agreements, and/or may be the same as the variables used in the fair shares approach. Like the planetary boundary control and response variables (section 1), the control variable should be quantifiable and can be influenced by human actions, while the response variable should describe stable conditions for the local water system and be influenced by the control variable. In some cases, the local safe operating space may represent aggregated impacts across multiple subjurisdictional locations. For example, defining the local safe operating space for a city may require managing water in multiple watersheds. In this case, the local safe operating space may require individual control variables corresponding to each of subjurisdictional hydrological functions (e.g., sufficient surface water availability in each watershed), or the local safe operating space could be defined using an aggregated approach (e.g., total available water for withdrawal).

Past attempts to define local safe operating spaces have typically used consumptive freshwater use, the same control variable as the water planetary boundary (Table S1; Cole et al., 2014; Fanning & O'Neill, 2016; Teah et al., 2016). However, the local safe operating space approach may require alternative or additional control and response variables based on the unique conditions of the local water system context. This flexibility is well aligned with multiple water sub‐boundaries corresponding to different water stores (Table 1). For example, in an analysis of regional safe operating spaces for two regions in China, Dearing et al. (2014) did not include a freshwater use boundary because the primary regional water challenges were related to water quality and sedimentation, rather than water quantity.

#### 2.2.2 Setting the Local Boundary

For operational purposes, setting a boundary value requires a defined relationship between a control variable and the response variable. In local contexts, the stable conditions of the response variable may be defined using the observed range during the Holocene (as in the water planetary boundary) or using other locally relevant environmental thresholds. Variables and boundaries identified in the safe operating space approach may not be the same as existing local management thresholds and will likely be more restrictive in areas that have experienced degradation of the hydrological cycle such as the Cienega Grande de Santa Marta wetlands (Box 1). For instance, Dearing et al. (2014) use historical measurements of hydrological and ecological variables to identify local boundary values. Local (and global) boundaries may be characterized by nonlinear relationships between the control and response variables, potentially including tipping‐type or hysteretic behavior, which can make it highly unlikely that the system will go back within the boundary once transgressed (Bauch et al., 2016; Foufoula‐Georgiou et al., 2015). As a result, once a local threshold is transgressed in a hydrological system, the control variable value required to reenter the local safe operating space may be significantly lower than the original boundary value due to negative feedbacks associated with hysteresis preventing transitions back to the original state (van Nes et al., 2016). Therefore, potential water regime shifts such as those reviewed in Falkenmark et al. (2019) can be identified to define locally relevant ranges of the response variable and corresponding control variables.

Using the planetary boundaries framework to define a local safe operating space has primarily been an academic exercise to date (Table S1), but for practical water management and governance, socioeconomic and equity concerns will come into play. During the setting of boundary values, both the characterization of stable hydrological conditions and assessments of acceptable levels of risk are likely to vary among stakeholders within a community as well as across local contexts. For example, poorer stakeholders may be less resilient to short‐term hydrological variability (i.e., define stable conditions as a narrower range in Figure 1) and have fewer options to reduce exposure to risk (i.e., set the boundary further back from estimated thresholds in Figure 1). Community‐level involvement has not been prioritized in past efforts to apply the planetary boundaries framework, but from a sustainable development perspective, local boundary setting can be rooted in environmental justice to define a “safe and just operating space” (Dearing et al., 2014; Leach et al., 2013; Raworth, 2012). This would require that all communities within the local context, not just the historically advantaged groups in a position of power, are meaningfully involved in defining and regulating the local safe operating space (Martín‐López et al., 2019).

#### 2.2.3 Quantifying the Current Value of Each Control Variable

Quantifying the current value of the control variable and comparing it to the local boundary value informs whether the local context is within its local safe operating space. Where data are available to define the relationship between the control and response variables, the current value of the control variable can be quantified in a fairly straightforward manner (Dearing et al., 2014). In many cases, however, data and deep understanding of the system needed to accurately estimate control variable values are lacking for some or all local boundaries. This may provide an opportunity to further integrate local communities in the definition of the local safe operating space. In addition to quantifying biophysical limits for a portion of the Heihe River in China, Teah et al. (2016) surveyed local residents on their perceptions on the current status of the control variables and the potential impacts of regional boundary transgression, finding that resident perceptions of the values of the control variables relative to their regional boundaries were mostly consistent with the quantified values. While the social survey was not used to set boundaries, it does indicate that local stakeholder involvement has the potential to both identify relevant control variables, estimate the present value for control variables where monitoring data do not exist, and evaluate the potential impacts of transgression.

## 3 Harmonizing Approaches to Integrate Local to Global Water Sustainability Targets

The fair shares and local safe operating space approaches each have benefits and drawbacks. The local safe operating space approach quantifies local limits to water system modifications but does not provide any information about potential external impacts beyond the local context being considered. The fair shares approach complements the local safe operating space approach by providing a tool for systematic comparisons among regions or countries, assessing global responsibility, and allocating responsibility for local contribution to global processes. However, the fair shares approach does not provide any guidance for whether the water cycle remains within locally important limits, which is the primary concerns of water managers and policymakers, and therefore requires integration with the local safe operating space approach. To take advantage of the strengths of each of these two approaches, we propose a methodology to harmonize the fair shares and local safe operating space approaches to develop a set of local boundaries that are consistent with both local and global water sustainability.

### 3.1 Harmonizing Fair Shares and Local Safe Operating Space Approaches

**Figure 3:**
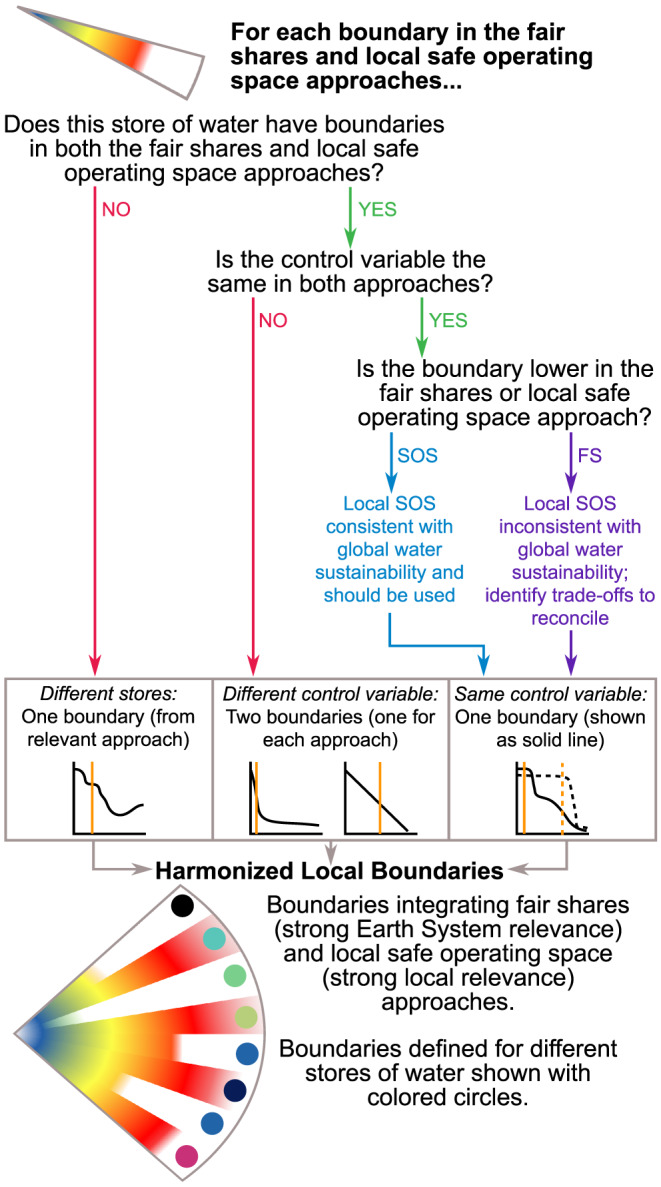
Decision tree for harmonizing the fair shares and local safe operating space approaches. Each sub‐boundary in the fair shares and local safe operating space approaches should be evaluated. The hypothetical plots corresponding to each type of boundary show the relationship between a control variable and a response variable as in Figure 1, and the orange line shows the boundary value. Each of the colored circles indicates a water sub‐boundary corresponding to a specific store of water from either the fair shares approach or the local safe operating space approach, as in Figure 2. The red‐yellow‐blue color gradient corresponds with the current position of the control variable with respect to the boundary, as in Figure 1.

For a given boundary, there are three potential relationships between the fair shares and local safe operating space approaches (Figure 3):

1. *Different stores:* For stores of water that are relevant in only one of the two approaches, no harmonization is needed since there will only be a single boundary value. For example, frozen water is unimportant in many tropical catchments and would be ignored in the local safe operating space approach but still considered in the fair shares approach due to its impact on global sea level. One could also envision a situation where a locally important store of water—for example, the water level in a lake—would be considered in the local safe operating space approach but not in the fair share approach due to its insignificant global impact. In this case, the local boundary could be defined based on the change in lake storage that would lead to a collapse of the aquatic food web (AghaKouchak et al., 2015; Kraft et al., 2012). In both examples, the control variable, response variable, and boundary value from whichever of the two approaches is relevant can be used.
2. *Different control variable:* For stores where there are different core water functions at the local and global scales, the control variables may differ between the fair shares approach and the local safe operating space approach. For example, the primary function of groundwater globally is providing baseflow to rivers during dry periods to maintain environmental flow requirements (Table 1). For the fair shares approach, this suggests a potential control variable of stream‐aquifer flux, a response variable of aquatic biosphere integrity, and a boundary value based on global environmental flow requirements (Gerten et al., 2013; Gleeson et al., 2019, 2020). However, in some local contexts, the presence of groundwater‐dependent terrestrial ecosystems suggests a potential control variable of groundwater depth below the land surface, a response variable of terrestrial biosphere integrity, and a boundary value when groundwater drops below the rooting depth (Eamus et al., 2015; Qiu et al., 2019; Rohde et al., 2017). For this type of relationship, a harmonized approach would require a unique set of sub‐boundaries for this water store, with separate control and response variables for each of the two approaches (i.e., fair share and local safe operating space).
3. *Same control variable:* For stores where the same control variable is used in the local safe operating space and fair shares approaches, the relationship between the control and response variables may not be the same in the two approaches. Modifying our hypothetical example for the groundwater sub‐boundary from the previous type, if the control variable is stream‐aquifer flux and the response variable is aquatic biodiversity for both the local safe operating space and fair shares approaches, the boundary value may be different in the two approaches if small changes in aquatic biodiversity would transgress the local safe operating space, for example, degrading a local fishery, without a negative impact on Earth System function. Where the local safe operating space boundary is lower (more environmentally conservative) than the fair shares boundary prioritizing local management will be consistent with global Earth System stability, and therefore, the local safe operating space boundary should be used. However, in cases where the local safe operating space boundary is higher (less environmentally conservative) than the fair shares approach, upscaling locally acceptable water management practices to the planetary level risks Earth System destabilization. In this case, the fair shares boundary should be used. Ethical and socioeconomic considerations are necessary to reconcile this mismatch between scales (Häyhä et al., 2016). For example, one may want to analyze trade‐offs to assess whether excessive local impacts can be compensated for by conservation elsewhere (section 3.2).

Based on these three types of relationships, the locally harmonized water boundary will always have at least as many sub‐boundaries as the water planetary boundary and may incorporate additional sub‐boundaries derived from the local safe operating space approach. The harmonized local boundaries will thus always be consistent with both global and local water sustainability goals and provide a framework for determining whether local water management is consistent with local socioenvironmental processes and Earth System function.

### 3.2 Recognizing and Respecting Real‐World Complexity

Comparing the local safe operating space and fair shares approaches can provide valuable insight into the cross‐scale relationships between sub‐global water systems and Earth System function. For instance, Gleeson et al. (2020) suggest that there may be “keystone regions” where water cycle modifications have a disproportionate impact on Earth System function. In keystone regions, we hypothesize that the local safe operating space and fair shares approaches would have similar control and response variables and boundary values, since the degradation of the local water system would lead to outsize impacts at the global scale. To avoid the implication that local water cycle modifications outside of keystone regions are unimportant, harmonization with the fair shares approach as described above and existing management and governance approaches is essential. Thus, while this paper primarily focuses on disaggregation from global to local scales, comparison between the local safe operating space and fair shares approaches may also be useful for determining appropriate techniques to aggregate from local to global scales (CISL, 2019).

While the typologies presented in Figure 3 are comprehensive of all potential local‐global interactions, the complexity of the real world will introduce trade‐offs among water sub‐boundaries and other planetary boundaries across spatial scales, time scales, degrees of reversibility, stakeholders, and types of environmental impacts (Booth et al., 2016; Qiu et al., 2018; Rodríguez et al., 2006). In addition, boundaries in the real world may not be expressed as two‐dimensional plots as shown in Figure 1 but rather as multidimensional parameter spaces representing multiple interconnected ecohydrological processes. Groundwater withdrawals for irrigation may, for example, enhance local net primary productivity and food production but alter regional‐scale hydroclimate (spatial scale trade‐off; DeAngelis et al., 2010; Harding & Snyder, 2012a, 2012b), impair groundwater‐dependent ecosystems (stakeholder trade‐off; Barlow & Leake, 2012; Gleeson & Richter, 2017; Zipper et al., 2018, Zipper, Gleeson, et al., 2019), make groundwater resources unavailable for future generations (reversibility and time‐scale trade‐off; Butler et al., 2018; Wada & Bierkens, 2014; Whittemore et al., 2016), and increase cropland at the expense of other ecosystem services or planetary boundaries, such as biodiversity and biochemical flows (environmental impact trade‐off; Anache et al., 2019; Foley et al., 2005, 2011; Hanaček & Rodríguez‐Labajos, 2018; VanLoocke et al., 2017).

There are numerous existing frameworks, models, and tools for understanding interactions and managing trade‐offs with diverse approaches including cluster analysis, integrated (nexus) modeling, multicriteria analyses, and trade‐off curves (Cavender‐Bares et al., 2015; Deng et al., 2016; Heck, Hoff, et al., 2018, Heck, Gerten, et al., 2018; Hurford et al., 2014). While it is beyond the scope of this paper to address how the water planetary sub‐boundaries can be integrated with existing trade‐off analysis tools, we note that managing trade‐offs requires understanding interactions among the water sub‐boundaries and other planetary boundaries. This motivates further research on understanding cross‐scale feedbacks between local water systems, social‐ecological conditions, and the Earth System. Managing trade‐offs may require tools to mobilize nonlocal financial resources as incentive or compensation for foregone local benefits when contributing to global sustainability targets and thus help a region stay within both local and global boundaries. This can follow examples from the climate finance and deforestation domain such as the Amazon Fund (Forstater et al., 2013; Nakhooda et al., 2013) and direct country‐to‐country payment mechanisms such as the UN REDD+ program (Roopsind et al., 2019).

## 4 Opportunities for Complementing Existing Water Management Approaches

**Figure 4:**
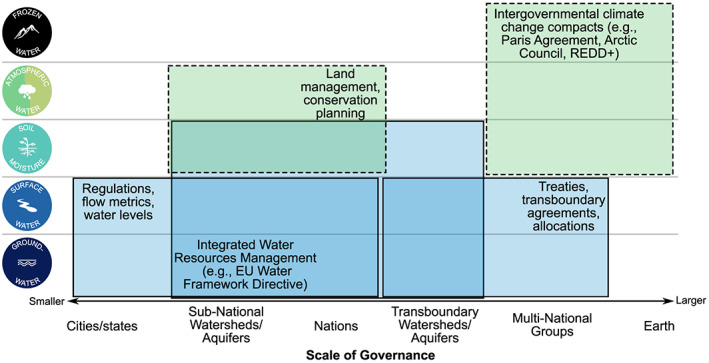
Examples of water management and governance approaches at different spatial scales targeting each store of water. Blue boxes are water management approaches, and green dashed boxes are management approaches that are not designed specifically for water but are likely to have a strong effect on that water store.

Numerous approaches to water management exist, most of which focus on either surface water or groundwater (Figure 4). In this section, we discuss how local applications of the water planetary boundary can complement these existing approaches (Table 2; CISL, 2019). Most importantly, the Earth System focus of the planetary boundaries demonstrates the necessity of expanding “water management” beyond the traditional focus on surface water and groundwater to include aspects of land management (related to atmospheric water, precipitation, and soil moisture) and climate change governance (related to changes in frozen water and in water availability). In all cases, the degree to which the planetary boundaries are adopted by stakeholders will depend on socioeconomic considerations, specifically the degree to which the perceived economic, social, and/or political benefits outweigh the perceived costs.

**Table 2:** Methods by Which the Water Planetary Boundary Complements Typical Water Management Approaches in Different Contexts

Context	Typical management approaches or metrics	Value added by the fair shares approach	Value added by the local safe operating space approach
Watershed or aquifer management (single jurisdiction or transboundary)	Flow metrics, groundwater levels, allocations, IWRM	• Integrates water with other Earth System functions, socioeconomic, and ethical considerations	• Integrates water with other Earth System functions, socioeconomic, and ethical considerations
		• Accounts for impacts outside the local context (global citizenship)	• Considers water fluxes beyond traditional water system boundaries
Political jurisdiction (state, national, or multinational)	Water policy and regulations, trade agreements, treaties	• Integrates water with other Earth System functions, socioeconomic, and ethical considerations	• Integrates water with other Earth System functions, socioeconomic, and ethical considerations
		• Accounts for impacts outside the local context (global citizenship)	• Considers water fluxes beyond jurisdiction boundaries
		• Provides consistency for comparing different countries or members	
Commercial organization (corporation or industry)	Life cycle analysis, industry standards, water footprinting	• Demonstrates commitment to global sustainability	• Evaluates resilience of supply chain
		• Provides consistency for comparing different companies or regions	

### 4.1 Watershed or Aquifer Management (Single Jurisdiction or Transboundary)

At the watershed or aquifer scale, existing management approaches often identify critical threshold(s) of streamflow, aquifer or reservoir water levels, or some other metric describing stores or fluxes of water. When watersheds or aquifers cross administrative boundaries, relevant metrics include water allocations to different stakeholders. These metrics often balance socioeconomic and biophysical considerations—for instance, how much water is necessary to support irrigated agriculture in the watershed while preserving sufficient in‐stream flows for aquatic ecosystems. Integrated water resources management (IWRM) is a watershed‐focused water management framework. Several core principles underlying IWRM and the planetary boundaries overlap: Both frameworks treat different stores of water as inherently interconnected and include land surface processes in the scope of water management (Badham et al., 2019). Since IWRM is designed to be applied at the watershed scale, the water planetary boundary provides a complementary framework for considering water flows that cross watershed boundaries through pathways including the following:

1. water use in the production of goods and services, i.e., through virtual water trade (Dalin et al., 2017; Marston et al., 2015; Zhang et al., 2020) or foreign direct investment;
2. flow through the atmosphere, i.e., moisture recycling (Wang‐Erlandsson et al., 2018, Keys et al., 2012, 2014, 2016), surface, i.e., transboundary rivers (Earle, 2013; Munia et al., 2016), or subsurface, i.e. regional groundwater flow (Ameli et al., 2018; Krakauer et al., 2014; Maxwell & Condon, 2016);
3. through physical infrastructure, i.e., interbasin water transfers (Garrick et al., 2019; McDonald et al., 2014); and
4. altered land‐atmosphere‐ocean interactions, i.e., local groundwater depletion leading to global sea level rise (Döll et al., 2014; Wada et al., 2010).

In particular, recent studies have shown strong regional‐scale connections across typical political and hydrological boundaries through atmospheric moisture flows. Globally, 57% of terrestrial evaporation eventually falls as precipitation over land, with substantial local‐ and regional‐scale variability (Van der Ent et al., 2010). For example, the majority of summer precipitation in some European watersheds is sourced from evaporation in other watersheds (Keune & Miralles, 2019), and Brazil supplies 13% to 32% of precipitation to other South American countries (Keys et al., 2017). These cross‐boundary evaporation‐driven fluxes indicate a need for integrating existing (blue) water management with domains typically thought of as land or climate governance, for example, via land use planning, and thus managing green water fluxes, within the planetary boundaries framework (Figure 4; Keys, Porkka, et al., 2019).

The European Water Framework Directive illustrates how the water planetary boundaries framework may complement existing watershed‐scale water management. The Water Framework Directive aims to reach and maintain “good” ecological and chemical status at the watershed scale, defined relative to natural conditions (Kallis & Butler, 2001). However, the Water Framework Directive does not establish or define any boundaries, nor does it situate this status relative to potential broader‐scale feedbacks with Earth System processes (Bishop et al., 2009). Thus, the water planetary boundary complements the Water Framework Directive in two primary ways. First, the fair shares approach to downscaling the water planetary sub‐boundaries provides a tool for assessing whether management actions in Europe are sufficient to maintain water stocks and flows and good ecological conditions from an Earth System perspective, especially through quantifying externalized environmental impacts via consumption‐based methods. Second, the local safe operating space approach provides a tool to set locally relevant boundaries within the context of the Water Framework Directive, which is particularly needed for heavily modified or artificial water features and to prioritize outcomes based on the local social‐ecological system. As of 2015, 47% of the European waters had not reached good ecological status, so it can be argued that the Directive has fallen short in delivering coherent and sustainable water management in Europe, reflecting the significant challenges for regional to global cooperation in water management (Voulvoulis et al., 2017). In sum, the water planetary boundary provides opportunities to better contextualize the Water Framework Directive at local and Earth System scales.

### 4.2 Political Jurisdictions (State/Province, National, or Multinational)

Political jurisdictions use a number of policies, regulations, and incentives to govern water, which are applied at numerous and often overlapping spatial scales (Wardropper et al., 2015). States/provinces, cities, or other jurisdictions may supplement national water‐related policies at subnational scales. Multiple nations can be bound together through political and trade agreements, including political units such as the European Union (EU) and African Union; trade agreements such as North American Agreement on Environmental Cooperation and the ASEAN Free Trade Area; and intergovernmental organizations such as the United Nations.

Chapron et al. (2017) argue the need for “legal boundaries” that translate the biophysical planetary boundaries into limits on human activities, designed and enforced to prevent transgression of planetary boundaries or to scale down the human pressures on boundaries that are already exceeded. At the scale of an individual political unit such as a nation, the planetary boundaries framework would provide a basis for considering the effects on stocks, flows, and processes of water external to the unit's borders, which activities within the unit may impact. Transboundary water management frameworks have been developed for some water sources (Eckstein, 2011; Puri & Aureli, 2005). However, these are unequally distributed globally and focus primarily on surface water watersheds and, to a lesser degree, aquifers (McCracken & Meyer, 2018). To our knowledge, no existing water management agreements address atmospheric water flows across watershed boundaries (Keune & Miralles, 2019; Keys et al., 2017) even though land use change or human water use can alter precipitation remotely (Keune et al., 2018; Wang‐Erlandsson et al., 2018; Zipper, Keune, et al., 2019). These transboundary atmospheric water flows indicate a need to expand the scope of water management beyond watershed and national scale and beyond blue water to include green and frozen water (see section 4.1; Creed et al., 2019).

Political jurisdictions are inherently limited in their ability to regulate outside their physical boundaries. The fair shares approach to planetary boundary downscaling is a tool that can be consistently applied in multiple locations, which can improve the equity and fairness of international agreements. A consistent quantification approach will aid in the adoption of local operational goals that are compatible with Earth System function, as there is increased likelihood of compliance among parties of agreements when the agreement is perceived as equitable and fair (Franck, 1988; Yihdego & Rieu‐Clarke, 2016). Intergovernmental organizations such as the World Trade Organization may facilitate the consideration of such cross‐border impacts. Additionally, this provides an approach to design agreements that address sustainability targets beyond their local geographic context, such as downwind and transboundary effects on water systems.

One example of how the water planetary boundary may contribute to water management beyond the national scale is provided by Häyhä et al. (2018). Taking a fair shares approach to downscaling, Häyhä et al. find that a consumption‐based approach to water use (e.g., water footprints) is essential to accurately calculate the EU's total impact on water systems, because >40% of water use caused by EU consumption of goods and services takes place outside its borders, mainly through agricultural imports. By systematically applying the same method across all countries in the EU, the authors are able to provide a consistent framework for interregional comparisons. Additionally, Häyhä et al. conclude that the primary benefit of the planetary boundaries framework relative to existing management approaches is the focus on interconnections between the water planetary boundary and other Earth System processes such as land system change and biogeochemical flows.

### 4.3 Commercial Organizations (Corporations, Industries, or Financial Institutions)

The water planetary boundary can also be used to guide decision making of commercial organizations. It may benefit a private‐sector stakeholder by providing traceable metrics for demonstrating a commitment to global sustainability and also assessing risk exposure along the value chain. Since the UN 2030 Agenda and Paris Agreement explicitly include the private sector as actors, there is a growing demand for the development of science‐based sustainability targets for commercial organizations, which consider negative impacts on environmental and hydrological resources and societal spillover effects. Clift et al. (2017) discuss the challenges and opportunities for businesses to use the planetary boundaries in their decision‐making strategies. In particular, they note that the planetary boundaries framework may provide a consistent approach to compare performance among different regions or companies using the fair shares approach outlined above (Table 2).

Effective use of the planetary boundaries can inform business decision making by highlighting the interdependence between economic activity and global sustainability. As long as full life cycle impacts on water resources are accounted for, as described in section 2, a corporation's fair share of the water planetary boundary could provide a globally consistent way to assess the water sustainability of a product or company, similar to a “fair trade” or “ocean‐wise” product branding (Butz et al., 2018). Since commercial organizations do not have physical borders (unlike watersheds/aquifers or political jurisdictions discussed previously), they may be more capable of effecting change in multiple jurisdictions via improved water sustainability actions. Additionally, corporations can use the water planetary boundary to identify risks to water along their global supply chain (CISL, 2019). A relatively small number of transnational corporations, deemed “keystone actors” as an analogy to the ecological concept of keystone species, have a disproportionate influence over some Earth System functions including marine ecosystems (Österblom et al., 2015), deforestation in boreal forests and the Amazon (Galaz et al., 2018), and more (Folke et al., 2019). Identifying and working with keystone actors provides one mechanism to significantly improve environmental outcomes, and such science‐business partnerships are currently emerging for global fisheries management (Österblom et al., 2017). Water risks and valuation frameworks have evolved from seeing water as a procurement cost, to understanding how water places assets and revenue at risk, to gaining an awareness of how water presents a strategic opportunity for value creation (Morgan et al., 2019). Existing frameworks such as the Alliance for Water Stewardship's International Water Stewardship Standard (AWS Standard) provide support for understanding sustainable water management within a catchment context but do not link to planetary boundaries and the interdependence between economic activity and global sustainability.

Several companies are exploring ways to implement the planetary boundaries framework, indicating the potential economic benefits of this framework from a commercial perspective. L'Oréal, a multinational beauty company, also include measures of freshwater ecotoxicity as well as water resource depletion (Vargas‐Gonzalez et al., 2019). Houdini, an outdoor clothing company, now includes a planetary boundaries assessment in its sustainability reporting (Haeggman et al., 2018). Alpro, a plant‐based foods company, has piloted the use of the planetary boundaries framework to set science‐based targets for nature and translate corporate activities into environmental impacts (Gladek et al., 2019). They propose accounting for blue and green water use at the basin scale (Gillespy et al., 2017). The Kering fashion company has partnered with the University of Cambridge to identify how businesses can best use the planetary boundaries framework for assessing corporate sustainability (CISL, 2019). This report highlights the differences between “downscaling” (i.e., fair shares approaches) and “upscaling” (i.e., local safe operating space approaches). CISL suggests that businesses should explore the opportunities of using the local safe operating space approach to guide corporate practices. Rather than trying to assess what is left to exploit, as might happen by comparing conditions to the global safe operating space under the fair shares approach, the focus should shift to actions needed to maintain and/or restore local environmental functioning in affected areas that would be identified using the local safe operating space approach. This is particularly important for globally heterogeneous boundaries like water because it considers hydrological impacts along the whole value chain, beyond the immediate local context.

**Box 1.** **Local‐global** **connections in the Cienaga Grande de Santa Marta Wetland Complex,** **Colombia**

**Figure 5:**
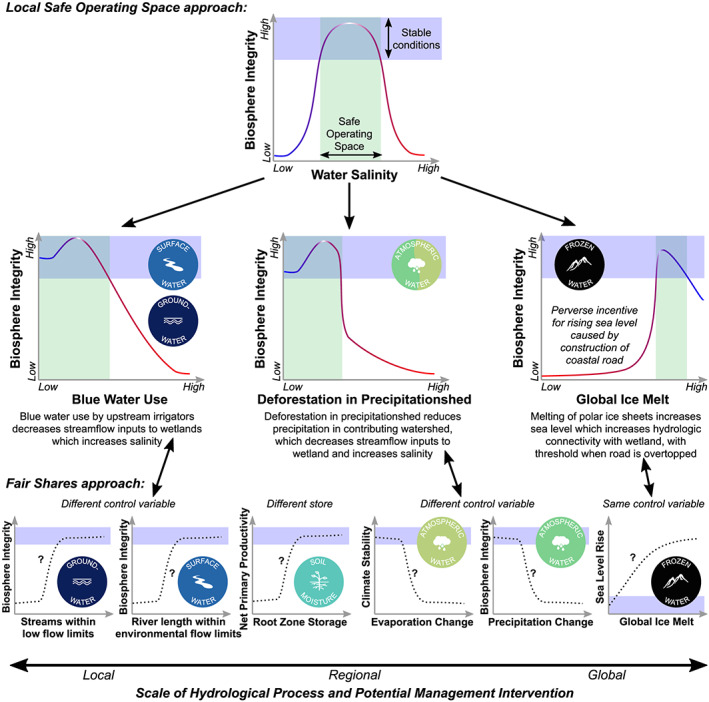
Qualitative definition of local safe operating space for Cienaga wetlands including underlying Earth System processes at local, regional, and global scales (top section) and the suggested control and response variables for the fair shares approach (bottom section). The lines on the local safe operating space plots show hypothesized relationships based on feedbacks described below the plots, and the lines on the fair shares plots are placeholders since global relationships necessary for downscaling are not yet known.

To demonstrate how the water planetary boundary may complement existing water management approaches, we present a case study of the Cienaga Grande de Santa Marta wetland complex in Colombia. This mangrove‐dominated system is susceptible to changes in salinity (Figure 5; Cardona & Botero, 1998; Elster et al., 1999), which is driven by the balance of freshwater inputs from precipitation and rivers, saltwater inputs from the ocean, and concentration of salinity via evapotranspiration within the wetland. Human activity has disrupted exchange between the rivers, ocean, and lagoon. Road construction in the 1950s cut off most surface water and groundwater exchange between the ocean and the wetland. In the 1970s and 1980s road and berm construction along the Magdalena River decreased freshwater inflows, leading to an increase in water salinity. Concurrently, irrigation and changes in land cover upstream led to a decrease in freshwater inputs and an increase in sediment loading to the wetland (Jaramillo, Licero, et al., 2018, Jaramillo, Brown, et al., 2018; Perdomo et al., 1998; Rodríguez‐Rodríguez, 2015). Beginning in the 1990s, the Colombian government developed a long‐term environmental management plan for the Cienaga wetlands (Botero & Salzwedel, 1999; Vilardy et al., 2011), focused on restoring hydrological and ecological conditions by mangrove reforestation and dredging to increase freshwater inflows (Figure 5). Mangrove cover has increased since the mid‐1990s (Jaramillo, Licero, et al., 2018), but recovery is slower than expected (Röderstein et al., 2014).

The planetary boundaries framework helps identify multiple feedback mechanisms occurring at nested spatial scales that define the local safe operating space of the Cienaga wetlands (Figure 5). The primary goal of local management in the Cienaga wetlands is protecting the mangrove ecosystem. For the local safe operating space approach, *biosphere integrity* (response variable) depends on keeping *water salinity* (control variable) within a narrow optimal range (Figure 5, top row). Three hydrological mechanisms occurring at three different spatial scales influence salinity and thereby bound the local safe operating space. At the local scale, freshwater inflows to the wetlands are influenced by upstream blue water withdrawals for intensive agriculture (Botero & Salzwedel, 1999; Vilardy et al., 2011). At the regional scale, the amount of incoming precipitation to the Cienaga wetland is influenced by ocean‐atmosphere cycles such as El Niño‐Southern Oscillation (Blanco et al., 2006; Hoyos et al., 2019; Restrepo et al., 2014) and also by terrestrial moisture recycling from the wetland's precipitationshed, which has areas of high deforestation (Keys et al., 2016; Zemp et al., 2014). At the global scale, sea level rise linked to ice sheet melt and climate change increases ocean‐wetland exchange. However, attention to the global scale alone could lead to a perverse incentive to allow increases in sea levels to improve wetland biosphere integrity, since sea salinity (~35 ppm) is lower than the current hypersaline conditions causing mangrove mortality (>100 ppm). These hydrological processes are additionally modified and affected by ongoing environmental change in the Cienega wetlands, for instance, upstream erosion and sedimentation associated with land use change reducing the hydrological connectivity between the river and the wetland (Botero & Salzwedel, 1999; Jaramillo, Brown, et al., 2018).

Applying the fair shares approach would apportion the water planetary sub‐boundaries to the Cienaga wetlands. Using the sub‐boundaries tentatively proposed by Gleeson et al. (2019, 2020), we see a mixture of relationships between the local safe operating space and fair shares approaches. The surface water and groundwater sub‐boundaries have different control variables. For the fair shares approach, the control variables primarily focus on in‐stream conditions (environmental flows and low flow thresholds), while in the local safe operating space approach the control variable is concerned with net inflows into the wetland lagoon, which is a function of blue water use. The soil moisture sub‐boundary is only relevant in the fair shares approach because local net primary productivity is not strongly dependent on soil moisture since the mangrove wetlands are frequently inundated. The atmospheric water sub‐boundaries also have different control variables in the two approaches: The fair shares approach uses changes in evaporation and precipitation change as control variables, and the local safe operating space approach uses deforestation in the precipitationshed of the contributing watershed. Finally, the frozen water sub‐boundary has the same control variable (global ice volume) for both the fair shares and local safe operating space approaches, but the local safe operating space creates a perverse incentive for rising sea levels due to the historic construction of a coastal road. This mismatch indicates that the local safe operating space approach for frozen water is inconsistent with global water sustainability (Figure 3), and thus, only the fair shares approach would be used for defining the safe operating space for frozen water in the Cienaga.

Combined, this analysis reveals several insights unaccounted for in current management efforts. First, the local safe operating space approach in particular can be used as a tool for setting limits to modification and targets for restoration of the local water system. Second, all aspects of the water cycle are important to the restoration of the Cienaga wetlands, though management focuses primarily on surface water and, to a lesser degree, groundwater (Vilardy et al., 2011). Third, accounting for atmospheric water and frozen water requires a broad and cross‐scale perspective that addresses drivers and risks at the local, regional, and global scales (Keys, Galaz, et al., 2019). In summary, the water planetary boundary provides additional useful insight into actions at multiple spatial scales from local to global that can help sustain the Cienaga wetlands. These nested spatial scales of Earth System processes and feedbacks among management and other factors indicate that collaborative, multiscale governance approaches will be necessary to halt or reverse the degradation of the Ciénaga wetlands.

## 5 Further Development of the Water Planetary Boundary to Complement Current Water Management and Governance Approaches

While the planetary boundaries framework is recognized to have value in policy integration and coherence, the studies we cite have generally remained as points for science‐policy discussion rather than actual policy shifts. Here we highlight three research priorities that will enable better integration of the planetary boundaries framework with existing water management and governance approaches.

First, the planetary boundaries framework can be regarded as a snapshot of a complex adaptive system. To integrate the planetary boundaries framework with water management and governance, it is essential to consider not just the current value of the water sub‐boundaries but also their temporal dynamics and the current values, trajectories and potential systemic changes of the other boundaries, over the course of a policy‐relevant time frame. In addition, changes in planetary boundaries are likely to have knock‐on effects on socioeconomic dynamics, with potential feedbacks influencing the water sub‐boundaries (Crépin et al., 2017). Each of the planetary boundaries is defined in terms of Holocene‐like conditions, which means that the control variables reflect the predominant interconnections and feedbacks of the Holocene Earth System, but these properties are changing in the Anthropocene, in particular because socioeconomic feedbacks play an increasing role in the trajectory of the Earth System (Waters et al., 2018). Policies based on water planetary boundary quantification should investigate not just the current status of a control variable but also its trajectory and relationship to the temporal dynamics of other ecological and socioeconomic components (Dearing et al., 2014). Integrating the planetary boundaries into management frameworks will drive the creation and adoption of new data analytics and modeling tools that can better handle the complex behavior of water in the Earth System, where it plays multiple physical, ecological and biogeochemical roles (Chang et al., 2018; Fan et al., 2019; Milly et al., 2014; Sippel et al., 2015).

Second, accounting for scientific uncertainty is a longstanding challenge for water management (Badham et al., 2019; Merz et al., 2015; Poff et al., 2016; Varela‐Ortega et al., 2011; Wei et al., 2011). Given the complexities inherent in Earth System dynamics, numerous aleatoric and epistemic uncertainties are embedded in the planetary boundaries framework. These will need to be estimated and communicated to policymakers and others (Westerberg et al., 2017) including (i) uncertainty regarding the complex and nonlinear feedbacks in the Earth System (Steffen et al., 2018); (ii) uncertainty regarding the relationship between the control and response variables at both local and global scales; (iii) uncertainty inherent in both observational data and models used to quantify the current value of the control variable (Long et al., 2014; Sperna Weiland et al., 2015); (iv) uncertainty in the approaches for aggregating/disaggregating control variables and allocating the safe operating space globally (Mace et al., 2014); and (v) uncertainty in the harmonization process of the two approaches here discussed. These uncertainties will most directly manifest in the definition of stable and unstable conditions for the response variable and integrating uncertainty into water management and governance, particularly aleatoric uncertainty, which is inherently unpredictable (Poff et al., 2016). The precautionary principle suggests that, as uncertainty increases, so should the setback from the estimated threshold value, particularly given the large impacts of crossing these thresholds (Crépin & Folke, 2015; Margolis & Nævdal, 2008) and the presence of substantial time lags (Crépin & Nævdal, 2019).

Third, the scientific community needs to work closely with policy and decision makers to transparently identify pathways that can meet existing local water management constraints while satisfying the Earth System sustainability defined by the water planetary boundary and indicate how performance on a harmonized approach will be evaluated. This will require international commitment to transdisciplinary fundamental and solutions‐oriented research, for example, through existing science‐to‐policy approaches and boundary organizations such as Future Earth (Suni et al., 2016). If we adopt the mindset that the planetary boundaries are guardrails defining a “corridor” that can be navigated through multiple sociopolitical pathways (Biermann, 2012; Qiu et al., 2018), changes to other planetary boundaries (e.g., land system change) may alter the effective size and shape of the hydrological corridor bounded by the water sub‐boundaries due to feedbacks between the water cycle and other components of the Earth System. As scientific understanding increases about these interactions and other changes in interrelated aspects of the Earth System, care needs to be taken to ensure that this shifting information baseline informs adaptive policy making rather than confounds it (Galaz, Biermann, et al., 2012). This further highlights the need for iterative approaches, such as adaptive management, that provide the opportunity to regularly update management strategies and targets (Gleeson et al., 2012; Pahl‐Wostl, 2007, 2008). In addition to its benefits, the planetary boundaries perspective may highlight new governance needs at both local and global scales (Biermann, 2012; Galaz, Crona et al., 2012; Galaz, Biermann, et al., 2012), for instance, fundamental incompatibilities between existing economic and environmental treaties (Biermann et al., 2012). But many of the integrated assessment models currently used for resource use assessments are essentially “black box” tools, where assumptions about human behavior, social structures, and economic priorities are invisible to the user. From an evaluation perspective, as more societal actors become engaged in water management, care needs to be taken to avoid “greenwashing”—creating the perception of water‐friendly practices without having tangible impacts on the control variable of interest. The harmonization of fair shares and local safe operating space approaches can help avoid greenwashing by providing ways to evaluate local management based on quantified changes in the value of a control variable, rather than practices intended or believed to have a positive benefit.

## 6 Conclusions

The planetary boundaries are a useful framework for defining the global safe operating space for humanity. However, the use of the planetary boundary for water management and governance at subglobal scales encounters major challenges in reconciling the global‐scale definition of the planetary boundary with the subglobal scales in which water decisions are made. Previous work to translate the water planetary boundary to local contexts has primarily adopted either a fair shares approach or a local safe operating space approach. The fair shares approach calculates the maximum allowable local contribution to the global planetary boundary and quantifies the global responsibility (contribution) of the local water use, while the local safe operating space approach uses the principles of the planetary boundaries framework to define locally relevant boundaries.

We present a harmonized approach to local use of the water planetary boundary that combines the advantages of the fair shares approach (Earth System relevance and global responsibility) and the local safe operating space approach (local relevance). This approach can be used in both socially defined contexts (cities, nations, companies, and industries) and physically defined contexts (watersheds, aquifers, and continents). Using these harmonized water sub‐boundaries will ensure that actions in a local context are contributing to water sustainability at all scales from local to global. Integrating the water planetary boundary with existing water management and governance approaches provides a framework to incorporate effects on water systems beyond the local, national, or watershed context; integrates socioeconomic and ethical considerations with biophysical constraints; and provides a consistent approach for interregional comparisons and quantification of the impact of water management solutions. Furthermore, the water planetary boundary further highlights the need for adaptive water management approaches that can respond to complex, nonlinear changes in Earth System processes.

## Supporting information



Supporting Information S1
