# Supplementary material for: Integrating the Water Planetary Boundary With Water Management From Local to Global Scales
Source: Earths Future. 2020 Feb 13;8(2):e2019EF001377. doi: 10.1029/2019EF001377 (PMC7375053; doi:10.1029/2019EF001377)
Supplement: Supplementary file 1 — Supporting Information S1 [file EFT2-8-e2019EF001377-s001.pdf]

Earth's Future

Supporting Information for

Integrating the water planetary boundary with water management from local to global scales

Samuel C. Zipper[1,2,*], Fernando Jaramillo[3,4], Lan Wang-Erlandsson[5], Sarah E. Cornell[5], Tom Gleeson[2], Miina Porkka[5,6], Tiina Häyhä[5], Anne-Sophie Crépin[5,7], Ingo Fetzer[5], Dieter Gerten[8,9], Holger Hoff[8,10], Nathanial Matthews[11], Constanza Ricaurte-Villota[12], Matti Kummu[13], Yoshihide Wada[14], Line Gordon[5]

1. Kansas Geological Survey, University of Kansas, Lawrence KS, USA
2. Department of Civil Engineering, University of Victoria, Victoria BC, Canada
3. Department of Physical Geography, Stockholm University, Stockholm SE-106 91, Sweden
4. Baltic Sea Centre, Stockholm University, Stockholm SE-106 91, Sweden
5. Stockholm Resilience Centre, Stockholm University, Sweden
6. Bolin Centre for Climate Research, Stockholm University, Stockholm, Sweden
7. Beijer Institute of Ecological Economics, Royal Swedish Academy of Sciences, Stockholm, Sweden
8. Potsdam Institute for Climate Impact Research (PIK), Member of the Leibniz Association, P.O. Box 60 12 03, 14412 Potsdam, Germany
9. Department of Geography, Humboldt-Universität zu Berlin, Unter den Linden 6, 10099 Berlin, Germany
10. Stockholm Environment Institute, Stockholm, Sweden
11. Global Resilience Partnership, Stockholm, Sweden
12. Instituto de investigaciones marinas y costeras "José Benito Vives de Andreis" – INVEMAR, Santa Marta, Colombia
13. Water and Development Research Group, Aalto University, Finland
14. International Institute for Applied Systems Analysis, Laxenburg, Austria

*Corresponding author: samzipper@ku.edu

Contents of this file

Table S1

Table S1. Previous use of the planetary boundaries in local contexts. Shaded rows indicate studies which did not define a water boundary.

Study	Context	Approach (all planetary boundaries)	Details related to water planetary boundary
Nykvist et al. (2013)	National (Sweden)	Fair shares: compare Sweden's performance to Rockström et al. (2009) boundaries using per-capita downscaling	*Control variable:* Consumptive blue water use<br><br>*Response variable:* Not stated<br><br>*Details:* Argue that fair shares approach not relevant for water since it does not account for regional variation in water availability
Cole et al. (2014)	National (South Africa)	Local safe operating space: develop local indicators and boundaries based on primary environmental concerns in South Africa	*Control variable:* Consumptive blue water use<br><br>*Response variable:* Biosphere integrity (implicitly by defining boundary based on local environmental flow needs)<br><br>*Details:* Quantified surface water availability after accounting for environmental flow needs, added estimated safe groundwater yield
Dearing et al. (2014)	Sub-national (two regions within China)	Local safe operating space: develop local control and response variables based on time-series data for primary environmental concerns	Water boundary was not defined - primary regional issues are water quality, air quality, and sedimentation.
Hoff et al. (2014)	National (all nations within European Union)	Fair shares: compare national performance to Rockström et al. (2009) boundaries using per-capita downscaling	*Control variable:* Consumptive blue water use<br><br>*Response variable:* Not stated<br><br>*Details:* Acknowledges limited utility of per capita downscaling, suggest future work should use context-specific factors such as environmental flows and also green water
Dao et al. (2015)	National (Switzerland)	Fair shares: compare national performance to Rockström et al. (2009) boundaries using per-capita downscaling with consideration of past and future generations	Water boundary was not defined - authors argue that only regional limits exist and global threshold not relevant.
Kahiluoto et al. (2015)	National (Finland and Ethiopia)	Local safe operating space: estimated local biochemical flows boundary based on historical data<br><br>Fair shares: per-capita downscaling of Carpenter & Bennett (2011) and Steffen et al. (2015) boundaries	Water boundary was not studied.

Sandin et al. (2015)	Industry (clothing in Sweden)	Fair shares: compare industry performance to Rockström et al. (2009) boundary using four ethical approaches	*Control variable:* Consumptive blue water use<br><br>*Response variable:* Not stated<br><br>*Details:* Compared four different ethical approaches to apportioning impacts. Note that framework could be adopted for regional, context-specific analysis.
Fanning & O'Neill (2016)	National (Canada and Spain) and sub-national (Nova Scotia and Andalusia)	Local safe operating space: calculate carbon, nutrient, water, and land footprint relative to local thresholds	*Control variable:* Consumptive blue water use<br><br>*Response variable:* Biosphere integrity, implicitly by defining boundary based on local environmental flow needs following Steffen et al. (2015).<br><br>*Details:* Use threshold of blue water consumption exceeding blue water availability for >3 months annually
Teah et al. (2016)	Sub-national (Middle reaches of Heihe River, China)	Local safe operating space: develop local indicators and boundaries based on primary environmental concerns in South Africa	*Control variable:* Consumptive blue water use<br><br>*Response variable:* Not stated<br><br>*Details:* Separately calculated low-risk and high-risk boundaries.
Brejnrod et al. (2017)	Individual building (comparison of two types of houses)	Fair shares: calculate performance of a single building relative to the per-capita carrying capacity	*Control variable:* Consumptive blue water use<br><br>*Response variable:* Not stated<br><br>*Details:* Primary water savings could be obtained by reducing living area per person.
Wolff et al. (2017)	Company (mass-market retailer)	Fair shares: Assess only one step in supply chain (agricultural production for food portfolio); full assessment of entire supply chain not feasible.	Water boundary was not defined - case study focused on biodiversity only.
Häyhä et al. (2018)	National (all nations within European Union)	Fair shares: compare per-capita and consumption-based downscaling of Rockström et al. (2009) boundaries	*Control variable:* Consumptive blue water use<br><br>*Response variable:* Not stated<br><br>*Details:* Suggest future work constrain freshwater use based on regional environmental flow limits
O'Neill et al. (2018)	National (all nations)	Fair shares: compare national performance to Rockström et al. (2009) boundaries using per-capita downscaling	*Control variable:* Consumptive blue water use<br><br>*Response variable:* Not stated<br><br>*Details:* Acknowledge limitations of per-capita downscaling

Ryberg et al. (2018)	Industry (laundry washing within European Union)	Fair shares: compare national performance to Rockström et al. (2009) boundaries using four downscaling approaches	*Control variable:* Consumptive blue water use<br><br>*Response variable:* Not stated<br><br>*Details:* Downscaling approaches based on economic indicators (consumption expenditure and gross value added). Choice of downscaling approach significantly affected results.

References in Table S1

Brejnrod, K. N., Kalbar, P., Petersen, S., & Birkved, M. (2017). The absolute environmental performance of buildings. Building and Environment, 119, 87–98. https://doi.org/10.1016/j.buildenv.2017.04.003

Cole, M. J., Bailey, R. M., & New, M. G. (2014). Tracking sustainable development with a national barometer for South Africa using a downscaled "safe and just space" framework. Proceedings of the National Academy of Sciences, 111(42), E4399–E4408. https://doi.org/10.1073/pnas.1400985111

Dao, Q.-H., Peduzzi, P., Chatenoux, B., De Bono, A., Schwarzer, S., & Friot, D. (2015). Environmental limits and Swiss footprints based on Planetary Boundaries. Geneva: Swiss Federal Office for the Environment (FOEN). Retrieved from https://archive-ouverte.unige.ch/unige:74873

Dearing, J. A., Wang, R., Zhang, K., Dyke, J. G., Haberl, H., Hossain, Md. S., … Poppy, G. M. (2014). Safe and just operating spaces for regional social-ecological systems. Global Environmental Change, 28, 227–238. https://doi.org/10.1016/j.gloenvcha.2014.06.012

Fanning, A. L., & O'Neill, D. W. (2016). Tracking resource use relative to planetary boundaries in a steady-state framework: A case study of Canada and Spain. Ecological Indicators, 69, 836–849. https://doi.org/10.1016/j.ecolind.2016.04.034

Häyhä, T., Cornell, S. E., Hoff, H., Lucas, P., & van Vuuren, D. (2018). Operationalizing the concept of a safe operating space at the EU level – first steps and explorations (Stockholm Resilience Centre Technical Report, prepared in collaboration with Stockholm Environment Institute (SEI) and PBL Netherlands Environmental Assessment Agency). Stockholm University, Sweden: Stockholm Resilience Centre.

Hoff, H., Nykvist, B., & Carson, M. (2014). "Living well, within the limits of our planet"? Measuring Europe's growing external footprint. Stockholm Environment Institute, 2014–5.

Kahiluoto, H., Kuisma, M., Kuokkanen, A., Mikkilä, M., & Linnanen, L. (2015). Local and social facets of planetary boundaries: right to nutrients. Environmental Research Letters, 10(10), 104013. https://doi.org/10.1088/1748-9326/10/10/104013

Nykvist, B., Persson, Å., Moberg, F., Persson, L., Cornell, S. E., & Rockström, J. (2013). National Environmental Performance on Planetary Boundaries (No. ISBN: 978-91-620-6576-8). The Swedish Environmental Protection Agency.

O'Neill, D. W., Fanning, A. L., Lamb, W. F., & Steinberger, J. K. (2018). A good life for all within planetary boundaries. Nature Sustainability, 1(2), 88–95. https://doi.org/10.1038/s41893-018-0021-4

Ryberg, M. W., Owsianiak, M., Clavreul, J., Mueller, C., Sim, S., King, H., & Hauschild, M. Z. (2018). How to bring absolute sustainability into decision-making: An industry case study using a Planetary Boundary-based methodology. Science of The Total Environment, 634, 1406–1416. https://doi.org/10.1016/j.scitotenv.2018.04.075

Sandin, G., Peters, G. M., & Svanström, M. (2015). Using the planetary boundaries framework for setting impact-reduction targets in LCA contexts. The International Journal of Life Cycle Assessment, 20(12), 1684–1700. https://doi.org/10.1007/s11367-015-0984-6

Teah, H. Y., Akiyama, T., San Carlos, R., Rayo, O. V., Khew, Y. T. J., Zhao, S., … Onuki, M. (2016). Assessment of Downscaling Planetary Boundaries to Semi-Arid Ecosystems with a Local Perception: A Case Study in the Middle Reaches of Heihe River. Sustainability, 8(12), 1233. https://doi.org/10.3390/su8121233

Wolff, A., Gondran, N., & Brodhag, C. (2017). Detecting unsustainable pressures exerted on biodiversity by a company. Application to the food portfolio of a retailer. Journal of Cleaner Production, 166, 784–797. https://doi.org/10.1016/j.jclepro.2017.08.057
